# Antiphospholipid Syndrome and Kidney Involvement: New Insights

**DOI:** 10.3390/antib5030017

**Published:** 2016-07-11

**Authors:** José A. Martínez-Flores, Manuel Serrano, Jose M. Morales, Antonio Serrano

**Affiliations:** 1 Department of Immunology, Instituto de Investigación Hospital Universitario 12 de Octubre, 28041 Madrid, Spain; martinezfloresja@gmail.com (J.A.M.-F.); mserranobl@gmail.com (M.S.); jmorales@h12o.es (J.M.M.); 2 Immunology Section, Universidad San Pablo-CEU, 28660 Madrid, Spain

**Keywords:** autoimmunity, antiphospholipid antibodies, end stage kidney disease, early graft loss, thrombosis antiphospholipid syndrome, aPL, B2GPI, IgA

## Abstract

Antiphospholipid syndrome is an autoimmune disorder characterized by vascular thromboses and pregnancy morbidity associated with antiphospholipid antibodies: lupus anticoagulant, IgG or IgM anticardiolipin or anti-beta 2-glycoprotein I. The kidney is one of the major target organs in antiphospholipid syndrome (APS). However, beyond the known involvement of the kidney in primary and associated APS, we may be observing a new form of APS within the context of renal failure. This review describes the classical kidney manifestations of APS and provides new considerations to be taken into account.

## 1. Introduction

Hughes syndrome, most commonly called antiphospholipid syndrome (APS), is an autoimmune multisystemic disorder characterized by thrombosis and pregnancy-related complications in patients with antiphospholipid antibodies (aPL) [1]. aPL comprise a heterogeneous group of autoantibodies directed against phospholipids or protein–phospholipids complexes [2,3].

The diagnosis of APS is made when one clinical and at least one laboratory criteria are fulfilled [4]. The international consensus statement updated in 2006 includes as clinical criteria one or more episodes of thrombosis, arterial or venous, in any tissue or organ. Clinical criteria must be confirmed by unequivocal techniques such as imaging studies or histopathology. Consensus APS pregnancy morbidity includes: (1) unexplained fetus death at or beyond the 10th week of gestation; (2) premature birth before the 34th week of gestation due to eclampsia, preeclampsia or placental insufficiency; and (3) three or more unexplained abortions before the 10th week of gestation.

Lupus anticoagulant (LA) in plasma, IgG and/or IgM anticardiolipin (aCL) in medium or higher titers (>40 GPL or MPL, or > the 99th percentile) and anti-beta-2-glycoprotein I (aB2GPI) antibodies (in titer > the 99th percentile) in serum or plasma determined in at least two occasions separated by at least 12 weeks comprises the laboratory criteria [4].

APS can be classified into four groups in accordance with the type of aPL presented by the patient [4]:

I—More than one laboratory criterion in any combination.
II a—LA present isolated.
II b—aCL IgG or IgM isolated.
II c—aB2PGI isolated IgG or IgM.

Although anti-B2GPI antibodies of IgA isotype were not included in the 2004-defined laboratory criteria for APS due to controversial results [5], researchers were encouraged to clarify their role in the APS in the same meeting [4]. The clinical importance of IgA aB2GPI has increased in the last few years [6,7] due to the utilization of kits useful to detect IgA aB2GPI [8] and the task force in the 13th International Congress on Antiphospholipid Antibodies (2010, Galveston, TX, USA) recommended testing for the IgA aB2GPI in cases negative for IgG and IgM and when APS is still suspected [9,10].

APS was first described by Hughes in the mid-1980s as a disorder of hypercoagulability in association with aPL [11]. This syndrome was first reported in a patient with systemic lupus erythematosus (SLE) [12]. However, shortly after APS was defined in this way, several authors suggested separate categories in order to group patients with APS. At present, there are three different APS disease entities: (1) Patients with APS criteria and without associated systemic autoimmune disorders are defined as having “primary antiphospholipid syndrome” (PAPS) [13,14]. This is currently the most common form of the disease [15]; (2) Patients with associated systemic autoimmune disorders such as SLE or rheumatoid arthritis (RA) are classified as having “associated antiphospholipid syndrome” (SAPS) [13,14]; (3) Patients with catastrophic antiphospholipid syndrome (CAPS), consisting in multiple organ thrombosis with simultaneous multiorgan failure and mortality rate near to 50% [14].

Although prevalence of aPL in healthy populations is between 1.0% [16] and 5.6% [17], the real prevalence of APS among the general population is unknown. The prevalence published in different works show significant variability [18] and APS is probably underdiagnosed. aPL are not exclusive to autoimmune diseases as they have also been detected in infections [19,20], neoplasias [21] or are chlorpromazine [22] or quinidine induced [23]. The estimated incidence of APS is approximately 5 cases per 100,000 persons per year [17].

It is unclear how thrombosis is developed in aPL. Some studies have associated aPL with different thrombotic pathways, including persistent activation of coagulation [24,25], platelet activation [26], vessel inflammation [27] or accelerated atherosclerosis [28].

Nevertheless, the presence of aPL is not the only condition to induce thrombosis formation. Patients with aPL for long periods of time need a “second hit” involving activation of innate immunity and a pro-inflammatory microenvironment to trigger thrombotic episodes. Infections or surgery have been suggested as a possible means of a second hit [14,29].

Establishment of consensus criteria for APS has made it possible to standardize patient groups but it has also generated controversy because these very restrictive criteria have limited use for clinical purposes [30]. Some authors have proposed a redefinition of APS [31]. In fact, manifestations associated with aPL antibodies such as heart valve disease, livedo reticularis (LR), some neurological manifestations such as migraines or epilepsy, stroke or thrombocytopenia were not included in the updated criteria despite some of them being frequent in APS patients [18,32].

Renal manifestations are one of the most prevalent events in APS patients and may co-exist with other conditions, especially with lupus nephritis. Renal involvement in APS is caused by thrombosis in any location within the kidney vasculature. Renal manifestations may include renal infarction, renovascular hypertension, APS nephropathy, thrombotic microangiopathy (TMA), renal artery stenosis and increased allograft thrombosis [33].

The true incidence of renal involvement in APS has not been well determined and may be underdiagnosed. This involvement may fluctuate between 9% and 10% of patients having APS [34]. The purpose of this review is to summarize the information about APS and renal involvement.

## 2. APS Nephropathy

APS nephropathy is clinically characterized by a syndrome of vascular nephropathy associated with proteinuria, hypertension and renal insufficiency [35]. Acute presentation of APS nephropathy includes TMA. TMA has distinctive patterns and is usually associated with nephortic range proteinuria [36,37], hypertension and renal failure [38]. aPL test is always a useful tool to distinguish whether TMA is caused by APS [38] instead of other entities such as hemolitic uremic syndrome or thtombotic thrombocitopenic purpura [39].

The most striking features of APS nephropathy are vasocclusive lesions (total or partial), including TMA [37]. Focal or diffuse microangiopathic areas that might affect any of the vessels within the renal vasculature, recanalizing thrombi in arteries and arterioles are other well-defined characteristics [36]. Nevertheless, other changes in biopsies have been described unrelated to thrombosis such as glomerular basement membrane reduplication, focal segmental glomerulosclerosis, intimal hyperplasia of arteries or acute tubular necrosis [36,37,40,41].

## 3. Systemic Hypertension

Hypertension associated with livedo reticularis was described by Hughes in the definition of APS. Systemic hypertension is considered a sensitive marker of nephropathy. Nochy et al. reported a prevalence of 93%, in some cases this being the only clinical sign suggesting nephropathy [37]. Cacoub et al. reported malignant hypertension in APS patients without SLE in five patients [42].

## 4. Renal Artery Lesions

Renal artery involvement can be uni- or bilateral and usually consists of occlusive lesions resulting from thrombosis. The most common clinical manifestation is renal thrombosis in the onset of severe hypertension [43].

Renal artery stenosis (RAS) is another characteristic condition in APS. A total of 26% of aPL positive patients with non-controlled hypertension were found to have renal artery stenosis, this being significantly higher than in hypertensive controls (8%) and healthy potential kidney donors [43]. Although the source of RAS is not clear, the response to anticoagulation suggests a thrombotic process [43,44].

## 5. Renal Vein Thrombosis

Thrombosis may occur in the main and/or minor renal veins of patients with PAPS and patients with associated SLE [45,46]. This manifestation has been particularly associated with LA positivity and is usually characterized by the presence of nephrotic range proteinuria [35,46].

## 6. Lupus Nephritis

APS nephropathy in SLE patients may vary from 11.4% to 39.5% according to several studies [47]. Silvariño et al. found that patients with LA plus IgG aCL had an increased prevalence of APS nephropathy, suggesting that these aPL may have a direct effect on the development of these lesions [48]. Furthermore, Zheng et al. found that LA accompanied by antibodies against B2GPI is related to glomerular microthrombosis, with a prevalence ranging from 20% to 30% [49].

## 7. Catastrophic Antiphospholipid Syndrome (CAPS)

CAPS is an accelerated variant of APS with multiorgan failure being described for the first time by Asherson [50,51]. Although many patients with APS have venous or arterial thrombosis within an isolated location in the body, a small proportion have undergone rapid onset of multiorgan thrombosis associated with high mortality [52].

Due to the low prevalence of this form of APS, estimated at 0.8%, there is little clinical experience regarding this form [15]. At least three organs must be affected in order to diagnose the CAPS form, and symptoms may develop within a few days or weeks. Although large vessels may be affected, more commonly there is thrombotic microangiopathy affecting small vessels in multiple organs. In 50% of cases, the heart, central nervous system, liver, skin and kidney are affected [53]. According to the CAPS registry data, at least 70% of the patients had renal involvement [54]. Death is usually the result of multiple organ failure in more than 50% in patients with APS [52,55].

It is noteworthy that approximately 60% of the CAPS are preceded by a precipitating event, mainly infections [35].

## 8. aPL in Hemodialysis Patients

End stage renal disease is a rare complication of APS and only a few studies have investigated this relationship. High prevalence of aPL in hemodialysis (Table 1) has been reported. These antibodies seem to be independent of age, sex, length of time on dialysis, drugs and chronic B and C hepatitis [56]. The mechanism by which these autoantibodies appear is not clear. However, dialysis membranes, trauma to blood in dialysis or infections have been postulated as possible causes [56]. Cardiovascular complications rank first among the causes of death in patients on dialysis with chronic kidney disease and end-stage renal disease and include thrombotic episodes in cerebral and myocardial vessels [57]. The presence of these autoantibodies is unclear because some studies have associated aPL with thrombotic events [58,59] but other studies have not [60,61]. Overall, the evidence with classic aPL is not clear regarding the pathogenic role in end stage renal disease.

## 9. aPL in Renal Transplantation

aPL in renal transplantation varies from 2% [34,67] up to 15% [68] of the patients. Sufficient evidence exists to show that aPL positive patients have a higher risk of developing thrombosis with consequent graft loss. Vaidya et al. reported six patients positive for aPL with renal graft loss by thrombosis in the first week of the transplantation [69]. Wagenknecht et al. found that 57% of patients with primary nonfunctioning renal allografts were aPL positive, the latter posing an important risk factor for early renal allograft failure [70]. However, some studies have not reported any association between aPL and risk of graft loss despite a high prevalence of pretransplant aPL [68]. The small number of patients included in the study may be the key to the non-association of aPL with graft loss.

It has also been reported that a minority of patients (15.7% of patients with aPL) can develop aPL post-transplantation. It is still unknown how patients can acquire aPL but it has been speculated that it may be caused by a post-transplantation infection [71]. Furthermore, the development of aPL after transplantation was associated with acute rejection [72].

## 10. The Role of IgA aB2GPI Antibodies in the Context of End Stage Kidney Disease

### 10.1. Dialysis

Patients with chronic renal failure have a high prevalence of cardiovascular problems, which are especially severe in patients on dialysis [73,74]. Cardiovascular complications are ranked first in cause of death in dialysis patients including thrombotic events in the brain and myocardium [57]. Endothelial dysfunctions are common in these patients and this may contribute to hypertension and arteriosclerosis [75].

As described previously, the use of IgA aB2GPI is not an accepted laboratory criterion for the diagnosis of APS. However, since Galveston 2010 IgA, aB2GPI testing has been recommended in patients with persistent clinical of APS and negative for consensus aPL [76].

In a prospective study, 124 patients on dialysis were followed-up for two years. Of these, 41 (33.1%) were positive for IgA aB2GPI and 83 negative. Of the 41 positive, 46.3% died during the follow-up while only 16.9% of the 83 negative patients died (*p* < 0.001). The main cause of death for IgA aB2GPI positive patients was cardiovascular. Survival in the group of IgA aB2GPI positive patients was significantly lower than in the negatives. The risk of death (Hazard Ratio) for IgA aB2GPI positive patients was 3.274 (for a confidence interval of 95%, *p* < 0.001) [77].

The prevalence of consensus aPL was aCL IgG (9.7%), aCL IgM (6.4%) and aB2GPI IgG (10.9%), however, consensus aPL were not related to mortality or vascular events. It has been speculated that antigens released from the membranes of endothelial cells interact with dialysis membranes complexed hapten, exposing new epitopes in hemodialysis patients [77]. These complexes could stimulate the formation of aB2GPI antibodies that can promote hypercoagulability and arteriosclerosis progression [78].

Another study with patients undergoing hemodialysis recently confirmed the high prevalence of IgA aB2GPI antibodies and their relationship to vascular complications [79].

Serrano et al. described a similar prevalence of IgA aB2GPI in patients undergoing peritoneal and hemodialysis, indicating that the proposed models of hapten-carriers complexes generated by B2GPI interaction with dialysis membranes and endothelial injury by dialysis system access to body could be discarded [80].

### 10.2. Renal Transplantation

Classic aPL in transplantation are scarce, however they seem to be a clear association with graft loss. Two studies have investigated the implications of IgA aB2GPI in renal transplantation. A 10-year follow-up prospective study was performed including all patients transplanted during the years 2000–2002 in the 12 Octubre Hospital. Pretransplant IgA aB2GPI was examined retrospectively in 269 patients. Eighty-nine patients (33%, group 1) were positive for IgA aB2GPI and 180 patients (67%, group 2) were negative. Group 1 had a higher incidence of early graft loss at six months (11.2% in Group 1 vs. 1.6% in Group 2; *p* = 0.002) the thrombosis of the vessels being the most prevalent cause of graft loss and was only observed in Group 1. In the multivariate analysis, IgA aB2GPI was an independent risk factor for the development of early graft loss and delayed graft function but was unable to prove IgA aB2GPI as an independent risk factor for thrombosis graft loss. Graft survival was similar in both groups after the first six months. Therefore, patients positive for IgA aB2GPI pretransplant have a high risk of early graft loss and a high risk of delayed graft function [41]. An extension of this study including 1375 patients made possible to relate IgA aB2GPI as an independent predictor of early graft loss due to thrombosis [81].

IgA aB2GPI antibodies drop immediately after renal transplantation and remain significantly lower than the levels observed in the pre-transplant status, even in patients who have lost their graft and have returned to dialysis [80]. This fact leads to a therapeutic doubt on whether immunosuppression can be effective in patients with APS, and suggests that further study is necessary.

How IgA aB2GPI induce early graft loss due to thrombosis is not clear but it has been speculated with surgery as a possible “second hit” triggering thrombosis [81]. This should be investigated by other groups because only one group has been described this relationship.

## 11. Treatment

There is no specific treatment for APS. Treatments administered to APS patients are primarily preventive. They consist in interfering with clotting mechanisms to prevent further thrombotic events. The drugs most used are unfractionated heparin (UFH), low molecular weight heparin (LMWH) and vitamin K antagonists (VKA).

UFH or LMWH are used in acute thrombotic events [82]. VKA anticoagulation is the treatment of choice in the long term, especially in patients with venous thromboembolism and embolic cerebral ischemia [83]. Treatment intensity is measured by the International Normalized Ratio (INR), which should range from 2.0 to 3.0 in these cases. The same treatment with or without low dose aspirin is indicated for arterial thrombosis [83]. Aspirin is indicated for patients with previous pregnancy morbidity with no thrombotic event. Results from observational studies have indicated a protective effect of aspirin in patients with asymptomatic SLE [84,85,86,87] and 197 women with APS [88]. Catastrophic APS should be treated with a combination of VKA, low dose aspirin and/or intravenous and/or plasma immunoglobulins [89]. Rituximab, hydroxycloroquine, plasma exchange, anti-C5 antibodies or statins can also be an alternative treatment for patients persistently positive for aPL [83,90]. Eculizumab is a complement inhibitor that has shown efficacy in preventing the recurrence of APS events after renal transplantation [91].

The development of new direct oral anticoagulant (DOAC) drugs has enabled new treatments. Dabigatran is a DOAC that has been shown to be effective in arterial thrombosis treatment [92]. Rivaroxaban is a selective inhibitor of factor Xa and has no effect on platelet activation [93]. Fondaparinux is a selective inhibitor of factor Xa. It has been used as a treatment for heparin-induced thrombocytopenia, but recently it has been found that Fondaparinux prevents binding of the B2GPI antibody to the B2GPI [94]. These anticoagulants do not require monitoring, which is a greater advance on anticoagulation. Nevertheless, because of its high price and its similar efficacy [93,95] with the current anticoagulants, it plays a secondary role.

## 12. New Perspectives

Despite treatments for APS, a multicenter prospective study of 1000 APS patients followed-up for 10 years concluded that it was necessary to search for new markers to prevent the complications of APS. Even though patients were under treatment, some of them continued to develop thrombosis [96]. Recently, a new method to detect specific circulating immune complexes (CIC) of IgA bounded to B2GPI (B2A-CIC) has been described [97]. The B2A-CIC identifies a subgroup of patients prone to develop thrombosis [98]. Testing of this B2A-CIC in the pretransplant population could provide a way to identify patients prone to graft loss. Other aPL such as anti-phosphatidilserine/prothrombin [99] or anti-annexin [100] may be helpful to identify new patients to be treated. It has been recently described that the mTORC pathway is involved in vascular lesions associated with the APS [101]. In this way, initial immunosuppression based on mTOR-inhibitors in renal transplant patients positive for IgA-aB2GPI-ab could be an alternative to the use of VKA to prevent thrombotic events.

## 13. Conclusions

APS is well recognized as an important cause of kidney injury, with specific clinical and histological features that may lead to renal injury caused by thrombosis at any location within the renal vasculature. Prompt evaluation should be performed in APS patients with manifestations such as systemic hypertension, livedo reticularis, hematuria, proteinuria or renal insufficiency without any other justifying etiology. Testing for aPL must also be considered for patients with any of these manifestations, especially IgA aB2GPI antibodies, the latter representing about 30% of patients in dialysis and transplantation. APS has focused mainly on patients with SLE, but patients with SAPS and renal failure only represents 2%–5% in hemodialysis or transplantation. We may be witnessing a new form of APS based on IgA aB2GPI antibodies not related with autoimmune diseases, which should be studied in the upcoming years. For patients with SLE and positive for aPL, APS nephropathy, alone or associated with SLE nephritis, should be considered in order to help guide prompt therapeutic decisions that may help to prevent the development of renal failure.

Anticoagulation remains the main treatment for patients with renal disease caused by APS. Future studies may help to identify better therapeutic targets.

## Figures and Tables

**Table 1:** Prevalence of consensus aPL in hemodialysis patients.

Reference	Patients	LA (%)	aCL (%)	aB2GPI (%)
Quereda (Nephron, 1988) [62]	56	22		
Grönhagen (BMJ, 1990) [63]	146		26	
García- Martín (NDT, 1991) [33]	51	22	31	
Matsuda (Thr Res, 1993) [64]	39	33	31	
Brunet (Kidney Int, 1995) [56]	97	19	15.5	
Valeri (Clin nephrol, 1996) [65]	230		29	
Ducloux (Kidney Int, 2003) [66]	324		26.8	
García-Martín (doctoral tesis, 1999)	192	26	36	18
TOTAL	939	18.7	26	18
